# Discrete Element Analysis of Shape Effect on the Shear Behaviors of Ballast

**DOI:** 10.1038/s41598-023-42070-9

**Published:** 2023-09-08

**Authors:** Wenjie Hou, Ang Li, Weimin Song

**Affiliations:** 1 School of Highway, Chang’an University, Xi’an, 710064 Shaanxi People’s Republic of China; 2 School of Civil Engineering, Central South University, 68 South Shaoshan Rd., Changsha, 410075 Hunan People’s Republic of China

**Keywords:** Civil engineering, Computational methods

## Abstract

Railway ballast layer is an indispensable component of railway transport. Ballast morphology plays an important effect in ballast-sleeper interaction and the durability of ballast layer. In this study, four types of ballast with different morphological parameters were generated and discrete element method was adopted to investigate the direct shear behaviors. The initial packing states were studied by analyzing the porosities and the normal contact force distributions. The shear results were verified by the reported testing results. On the other hand, one-way ANOVA tests were performed to explore the impact of ballast shape on the initial packing behaviors and the direct shear performance. Results indicated that for all four parameters, aspect ratio (AR), sphericity (Φ), roundness (RD) and convexity (CON), the initial porosities decreased first and increased subsequently along with the increase of the parameters. The four parameters could significantly affect the internal friction inside the assemblies. For each parameter, the larger the parameter, the insignificant the internal friction effect. One-way ANOVA tests revealed that all the four parameters were significant in affecting the initial porosities and the internal friction. Moreover, during the direct shear process, the larger the AR or Φ, the smaller the coordination number, which was mainly ascribed to the ballast shapes.

## Introduction

In China, due to the huge transport volume and relatively low cost, ballasted railway, especially the heavy haul railway, has been playing an important role in the development of the national economy. The ballast layer is an indispensable component of ballast track system^1,2^. Ballast bed is directly underneath the sleepers, supporting sleepers, dispersing and transmitting the traffic loads to the sub-ballast layer and subgrade^3–5^. In addition to providing vertical support, the ballast imposes horizontal restraint on sleepers to maintain track alignment under the repeated traffic loading^6^. Moreover, a ballast layer provides the benefit of facilitating water drainage, thus ensuring the durability of the subgrade. However, under the repeated vehicles’ loading, particles’ movement and settlement may occur, which alter the contact condition between ballast and sleepers, and further result in a complex and unpredictable ballast-sleeper interaction^7^. These would pose detrimental effects on the operational safety and ride quality.

Ballast shape and gradation must comply with specific standards to ensure the quality and the deformation resistance to traffic loadings^8,9^. In these standards, the morphological parameters, such as flakiness index, shape index, particle length and gradation, are clearly addressed. Ballast morphology strongly affects the ballast degradation, which is critical in affecting the ballast’s behaviors. Ballast degradation introduces lots of fine particles, which is a phenomenon named as ballast fouling^10^. Fouling agent in ballast could mitigate the particles' interlock and decrease the friction angle, and in turn increases the deformation of rail tracks^11^. Guo et al.^12^ conducted the Los Angeles Abrasion (LAA) test on railway ballast, and found that the flaky or elongated ballast particles are susceptible to break in LAA test. After ballast degradation, the sharp corners in ballast tend to become more rounded. Huang and Tutumluer^10^ found that ballast fouling could lead to sleeper hanging. Meanwhile, the lateral resistance of ballasted track is reduced due to the inclusion of fouling agents^13^. Besides, the reduction effect on lateral resistance is closely related to the fouled position of ballast bed, and the ballast fouling in shoulder plays the greatest impact on the lateral resistance reduction^13^.

In the research of ballast behaviors and the ballast-sleeper interaction, discrete element method (DEM) shows unparalleled advantages in revealing the internal mechanisms which govern the ballast's performance. DEM could compute the velocities and positions of a huge number of particles, and also reveal the interactions between particles based on Newton’s second law^11,14–18^. In exploring the ballast behaviors using DEM, ballast shape is a main factor affecting the internal heterogeneity. Aspect ratio is generally employed to characterize the ballast shape. Gong and Liu^19^ found that as the aspect ratio increased, the ratio between the deviatoric stress and the effective mean stress also increased, and the ratio reached to the maximum value when the aspect ratio was 1.50. Deng and Davé^20^ found that when no cohesive force existed, a constant aspect ratio generated a constant porosity, and the porosity was independent on the particle size. As there are many parameters characterizing the shape, to characterize particle shape in a collective manner, Yang and Luo^21^ proposed a parameter of overall regularity (OR), which is the average of convexity, aspect ratio and sphericity. It was found that with the increase of OR, particles are more regular and rotund. It was also concluded that the critical state properties of the triaxial tests were sensitive to the variation of shape parameters. Jiang et al.^22^ added one more parameter, roundness, and regarded the average value of the four parameters as the overall regularity (OR). It was found that OR affected the response of the granular assemblies in terms of the stress–strain relations, dilatancy, and critical state behaviors. Bian et al.^23^ extracted many morphological indices by employing some image techniques. As the angularity index (AI) and flat & elongated (F&E) ratio increased, the particle interactions became stronger and the coordination numbers were larger. Xiao et al.^24^ proposed the concept of curvature index (CI), which was employed to describe the local morphological feature of a particle. The Fourier-based random generation method was also commonly used to generate particles with complicated morphologies, and then the macro- and micro-behaviors were investigated^25^. Danesh et al.^26^ found that by increasing the angularity index, the peak internal friction angle and dilation increased.

In generating ballast particles with realistic shapes, some scholars used a 3D laser scan to obtain the surface data of ballast, and then reconstruct the ballast in DEM using some algorithms^27–29^. For 2D simulations, image techniques could be used to obtain the profiles^30^. Clumps or clusters are then prepared to represent ballast particles with complicated shapes. Lu and McDowell^31^ stated that the interlocking provided by the clumps provided a much more realistic load deformation response than the spherical particles. The other method to produce ballast shapes is the numerical method. Liu and Ji^32^ created some dilated polyhedral ballast by the Minkowski sum theory. Some poly-superellipsoid particles based on the traditional superquadric equation were also generated^15,33,34^. These approaches provide convenience for studying the macro and mesoscopic behavior of real ballast.

The direct shear test (DST) is commonly adopted to study various behaviors of geotechnical materials, such as the internal friction, dilatancy behavior, stress–strain relations, and strength envelop parameters, etc.^28,35–38^ due to the advantages in the simplicity of the device, the simplicity of testing procedure and good data repeatability. The above mentioned literature review showed that although there existed some studies about the effect of shape on ballast behaviors, studies about the effect of the shape parameters on ballast meso-performance in a quantitative manner are still limited. In this study, we created some ballast with different morphological parameters based on a numerical rule, and then the direct shear tests were carried out to explore the effect of the shape parameters on the macro- and micro-behaviors of railway ballast. The initial packing behaviors of ballast with different shape parameters were explored. One-way ANOVA tests were also conducted to evaluate the significance of the shape parameters on the packing and shear behaviors.

## DEM modeling

### Material

**Figure 1:**
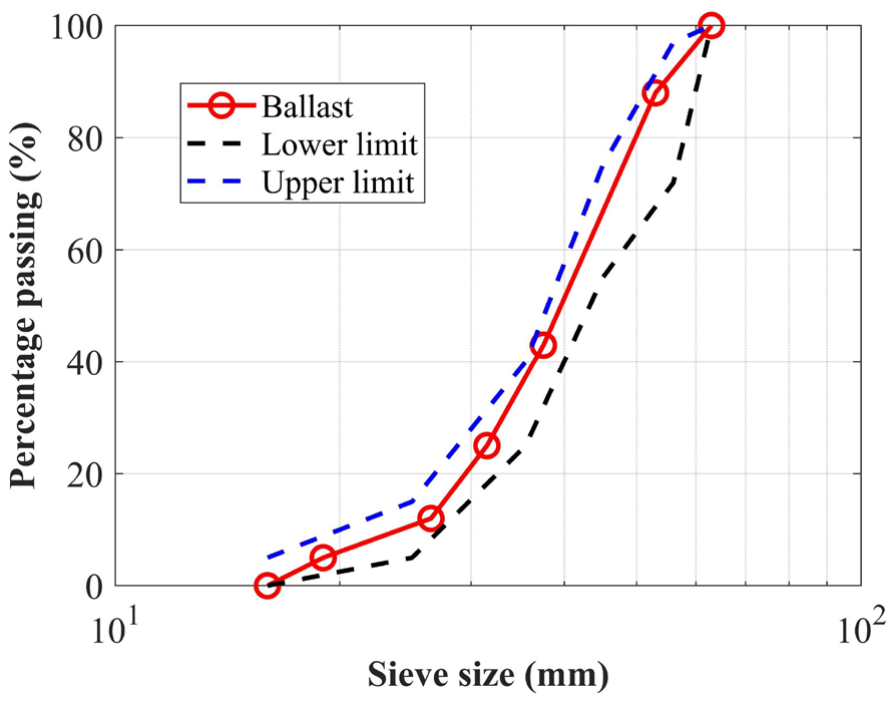
Ballast gradation.

**Figure 2:**
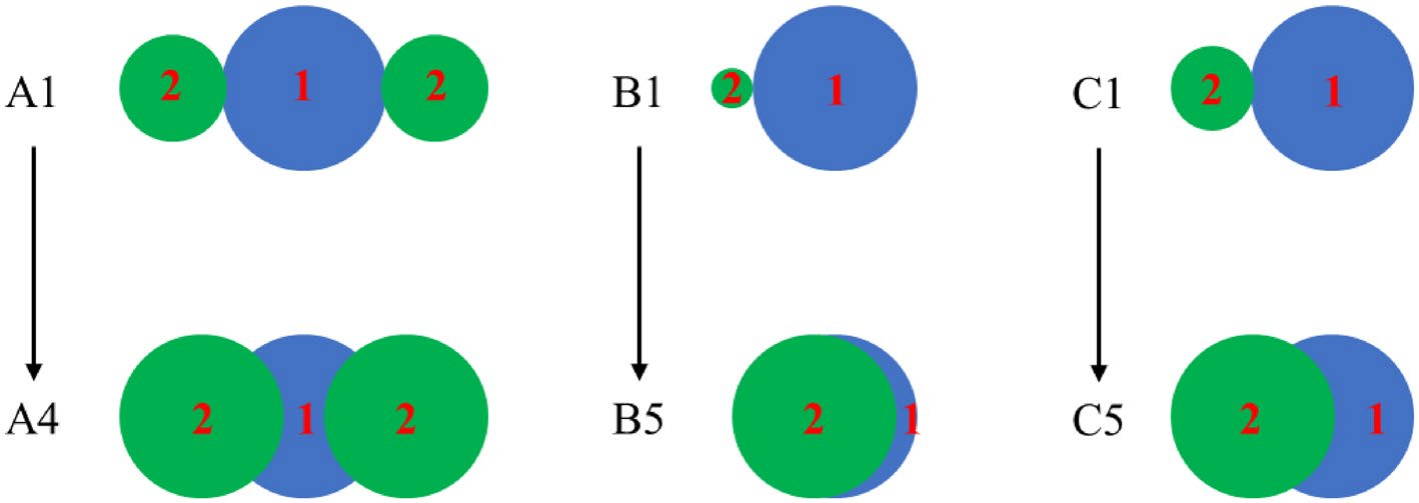
Three types of ballast generated by clump.

In this study, ballast gradation was determined based on the Chinese Standard TBT 2140–2008. Ballast gradation can be seen in Fig. 1. The maximum size of ballast is 63.5 mm. This study is to investigate the effect of particle shape on the direct shear performance of ballast. To produce different morphological parameters, four types of particles, A, B, C and D, were generated, in which A, B and C are clumps representing the relative complicated particles and D is spherical particles. In Fig. 2, for clumps—A, B and C, the major axis length of a clump was constant, and the size of particle 1 was constant, the morphological parameters can be modified by changing the locations and sizes of particle 2. From A1 to A4, B1 to B5 and C1 to C5, the size of particle 2 gradually increased and thus the volume of intrusion between particles 1 and 2 gradually increased. It should be noted that although there exists difference between the simulated particles and real ballast, the main purpose of this study is to reveal the effect of shape parameters on the shear behaviors of ballast from a meso-scale perspective. On the other hand, the generation of the particles in this manner in Fig. 2 is more convenient and less time-consuming.

**Figure 3:**
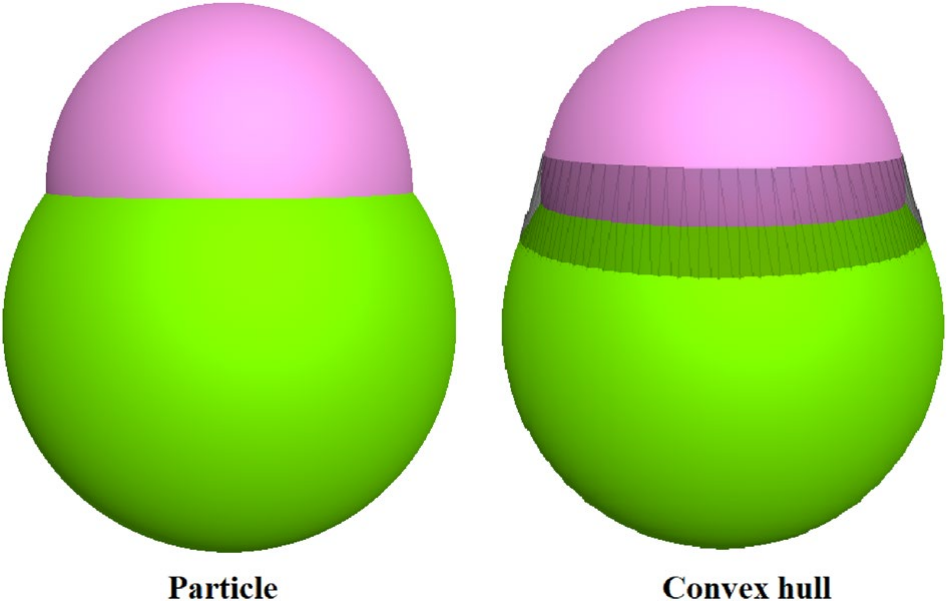
Convexity.

In this study, the parameters characterizing the ballast morphology include aspect ratio (AR), sphericity (Φ), roundness (RC) and convexity (CON). Aspect ratio (AR) is the average of the elongation index (EI) and the flatness index (FI)^39^. The calculation of EI and FI can be realized by acquiring the three representative axes of an object (P_1_, P_2_ and P_3_). In the three representative axes, P_1_ > P_2_ > P_3_, determined by the principal component analysis. Sphericity (Φ) is a parameter defined as the ratio of the nominal surface area to the actual surface area of an object^40^. However, due to the difficulty in calculating the areas, other calculation methods of sphericity are used based on length measurement of P_1_, P_2_ and P_3_^41^. Roundness (RC) is the ratio between R_2_ and R_1_, where R_1_ is the equivalent sphere radius of a clump and R_2_ is the minimum radius of a sphere imbedding the particle. Convexity reflects how closely a particle represents a convex hull. The convex hull is the smallest convex set that contains it. This parameter can be calculated using Eq. (4). The parameters of the four types of ballast are shown in Table 1.

1 $${\text{AR = }}\frac{1}{2}\left( {\frac{{{\text{P}}_{{2}} }}{{{\text{P}}_{{1}} }}{ + }\frac{{{\text{P}}_{{3}} }}{{{\text{P}}_{{2}} }}} \right)$$

2 $$\Phi { = }\left( {\frac{{{\text{P}}_{3}^{2} }}{{{\text{P}}_{{1}} \cdot {\text{P}}_{{2}} }}} \right)^{1/3}$$

3 $${\text{RC = R}}_{{2}} {\text{/R}}_{{1}}$$

4 $${\text{CON = V/V}}_{{{\text{ch}}}}$$

where P_1_ is the major axis length, P_2_ is the medium axis length, and P_3_ is the minor axis length, V is particle volume and V_ch_ is the volume of its convex hull, which can be observed in Fig. 3.

**Table 1:** Morphological parameters.

	AR	Φ	RC	CON
Particle A1	0.722	0.763	0.514	0.771
Particle A2	0.722	0.763	0.517	0.785
Particle A3	0.722	0.763	0.600	0.951
Particle A4	0.722	0.763	0.641	0.966
Particle B1	0.9	0.928	0.679	0.956
Particle B2	0.9	0.928	0.687	0.959
Particle B3	0.9	0.928	0.749	0.979
Particle B4	0.9	0.928	0.85	0.99
Particle B5	0.9	0.928	0.888	0.999
Particle C1	0.833	0.874	0.571	0.876
Particle C2	0.833	0.874	0.587	0.884
Particle C3	0.833	0.874	0.661	0.916
Particle C4	0.833	0.874	0.751	0.942
Particle C5	0.833	0.874	0.840	0.989
Particle D	1	1	1	1

### Direct shear test (DST) modeling

**Figure 4:**
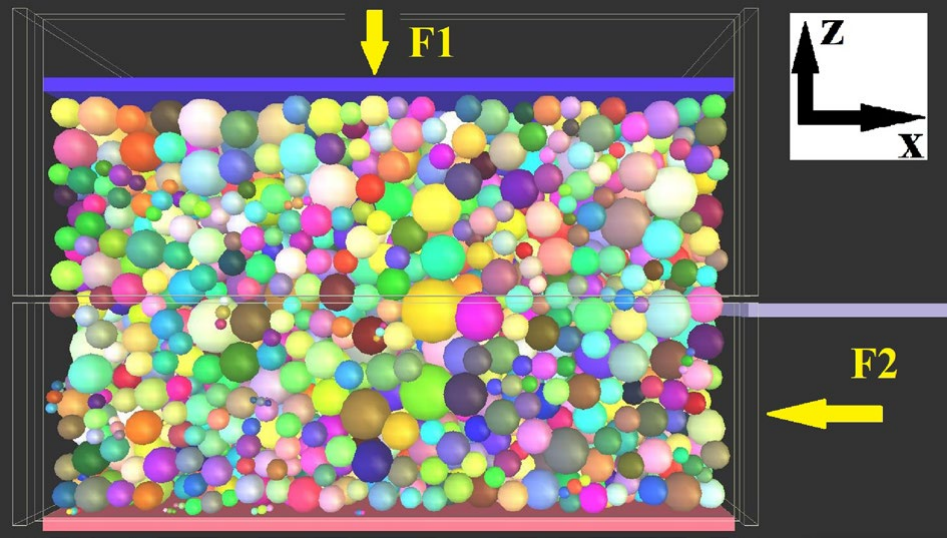
Shear test setup.

The DST models were conducted using a mould with the size of 500 × 500 × 350 mm. The size ratio between the chamber dimension and the maximum size of ballast is 7.9. As indicated by Indraratna et al.^42^, the box size in this study is large enough to neglect the boundary effect on the simulation results. Firstly, the ballast assemblies were generated in a loose state. Particles then fell under gravity and then formed an initial packing state. To prepare a compacted ballast layer, an external plate started to compact on the ballast. The load was applied at a frequency of 0.25 Hz for 80 s in the form of a sine wave, the magnitude of which was from 0 to the defined normal force. The normal stresses were set as 50, 100 and 150 kPa, respectively. The normal stress was applied on the top surface (F1 in Fig. 4) through a servo-control mechanism, which could adjust the velocity of the top wall until the reaction stress within the margin of error. Then, the direct shear test was carried out. Previous studies generally selected a low shear speed from 0.0167 to 0.2 mm/s to better control the normal stress fluctuation^23,43,44^. In this study, all the walls in the lower box were moved at a constant speed 0.0001 m/s along the negative axis, which can be seen in Fig. 4. Meanwhile, the movement of all the walls in the upper box, except for the top wall, were restrained. The tolerance limit of normal stresses was set as ± 2.5%. When the global shear strain (the relative displacement in negative x direction of the lower box divided by the length of the sample) reached 20%, the shear test was finished. For each assembly in Table 1, direct shear tests were conducted in triplet, and there are total 45 shear tests.

In the tests, the horizontal displacement, shear stress, normal stress and vertical displacement were recorded automatically. Shear stress is measured by tracing the reaction forces in the x direction on all boundaries in the underlying layer, and is calculated by the Eq. (5).

5 $$\tau { = }\sum\limits_{i = 1}^{5} {F_{Xi} } /A$$

where *F*_*Xi*_ is the reaction force in the x direction of the ith wall in the lower box, and *A* is the area of the shear interface.

The friction coefficient is in the range of 0.5–0.8^11,16,24,45^. In this study, a friction coefficient of 0.6 was selected to characterize the friction between ballast particles. Other parameters in DEM modeling are shown in Table 2.

**Table 2:** Parameters used in DEM modeling.

	Ballast	Box
Density (kg/m^3^)	2550	7800
Elastic modulus (MPa)	150	200,000
Poisson's ratio	0.2	0.25
Friction coefficient	0.6	0.5

In this study, the traditional linear elastic–plastic contact model^18^ was adopted in this study. The contact force is composed by two components: the normal force and tangential force. The Coulomb sliding criterion is also used together with the standard incremental algorithm to calculate the shear force. An open-source code Yade^46^ was used for all the DEM tests. Python is used for model construction, simulation control, and post-processing. Each DST test took about 4 h on an Ubuntu 18.04 Desktop (Intel Core CPU I7–8700, 4 cores at 3.4 GHz, 16 GB RAM). All the statistical analysis and figure plotting were conducted using Matlab.

## Results and discussion

### Packing behaviors

**Figure 5:**
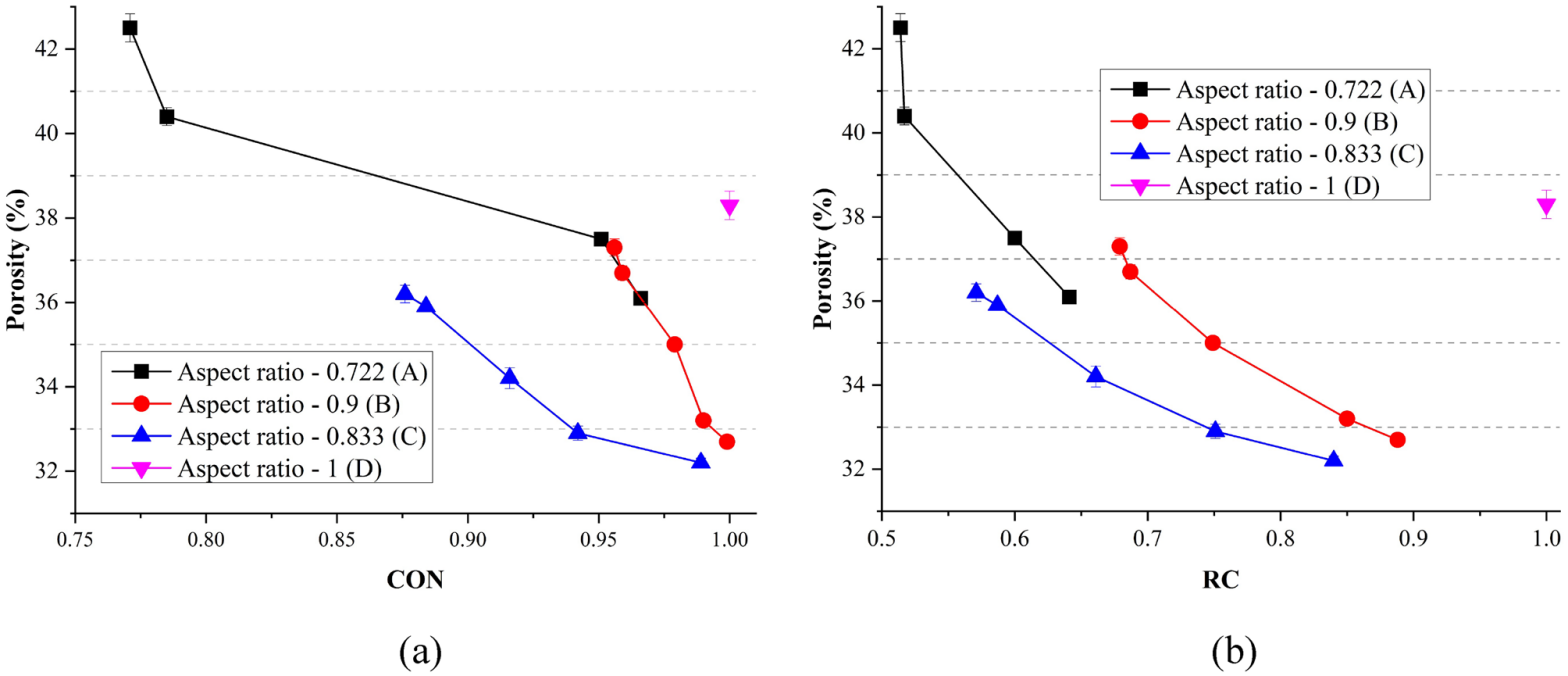
Porosities of all DST tests considering the development of: (**a**) CON; (**b**) RC.

Figure 5 gave the porosities of assemblies. For the same particle type, as the convexity (CON) increased, the porosities decreased obviously. With the increase of CON, less addition volume was needed to form a convex hull. During packing, the recessed volume generally cannot be filling properly; thus, the smaller the CON, the larger the porosity. It should be noted that although CON of particle D equaled to 1, the porosity of D assembly was not the least. Same conclusion could be made on the effect of RC on the porosity. For the same type of particle, the larger RC, the larger porosity.

**Figure 6:**
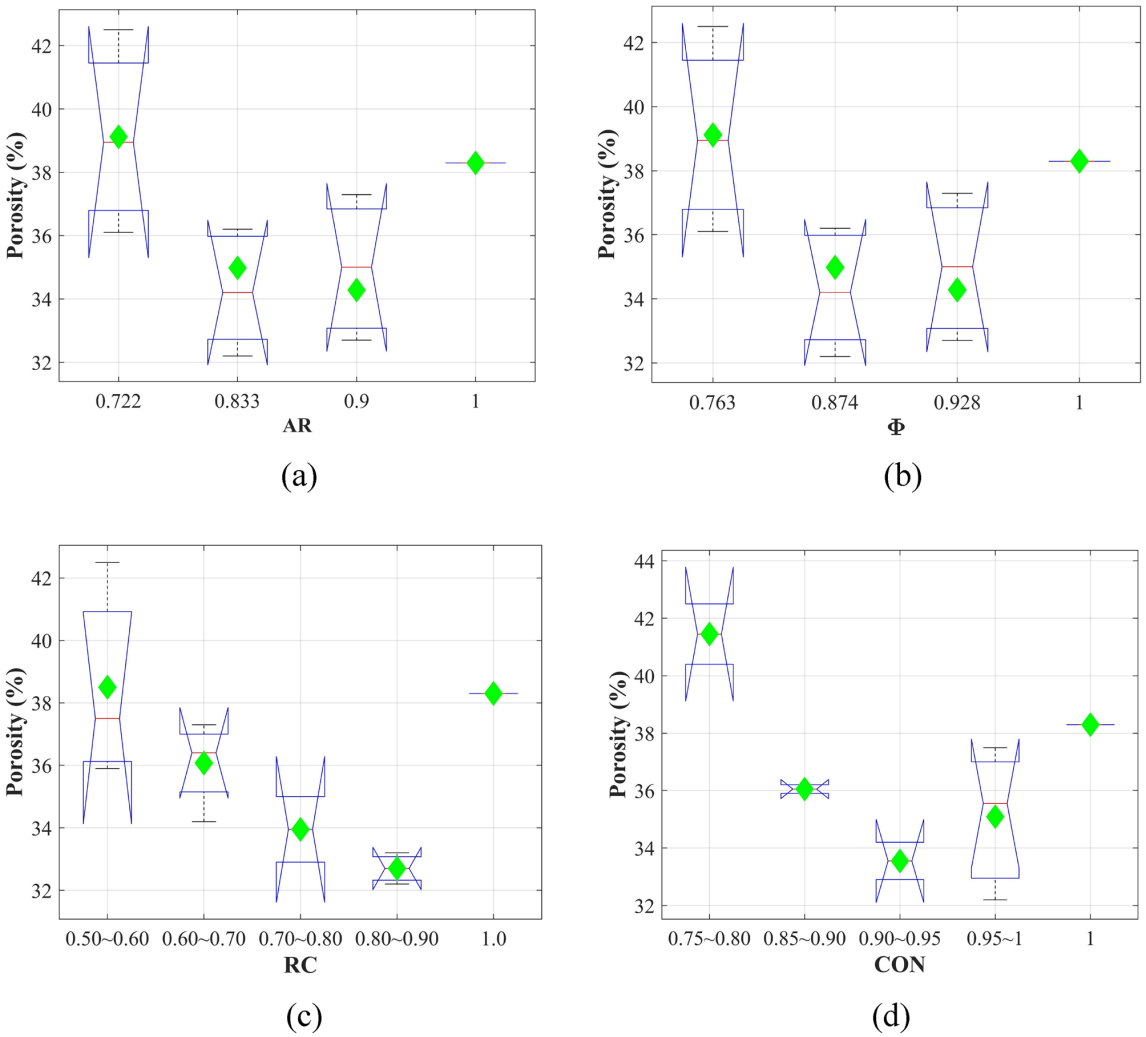
Porosity distributions for a single parameter: (**a**) AR; (**b**) Φ; (**c**) RC; (**d**) CON.

Figure 6 presents the porosities of ballast assemblies considering different parameters. In these figures, the central mark is the median value, and the 25^th^ and 75^th^ percentiles of the data are shown as the edges of the box. The green diamonds present the mean values of different groups. Based on the ballast generation approach, when AR and Φ are both constant for different type of ballast, the porosity distributions are same for parameters AR and Φ. For RC and CON, several groups were divided, which are shown as the horizontal axes in Fig. 6c,d. It can be observed that when AR equals to 0.9 and Φ equals to 0.928, the porosity could obtain the minimum value. For RC, RC in the range of 0.8–0.9 could make the packing denser. When CON is between 0.90 and 0.95, the porosity was the minimum. For all four parameters, the porosity decreased first and increased subsequently along with the increase of the parameters. Table 3 shows the one-way ANOVA results of the porosity. All parameters (AR, Φ, RC, CON) were all significant factors at the 95% confidence interval.

**Table 3:** One way ANOVA tests of porosity.

	Source	SS	df	MS	F	Prob > F
AR	Groups	64.266	3	21.422	4.36	**0.03**
	Error	54.063	11	4.915		
	Total	118.329	14			
Φ	Groups	64.266	3	21.422	4.36	**0.03**
	Error	54.063	11	4.915		
	Total	118.329	14			
RC	Groups	77.557	4	19.389	4.76	**0.021**
	Error	40.773	10	4.077		
	Total	118.329	14			
CON	Groups	83.286	4	20.821	5.94	**0.01**
	Error	35.044	10	3.504		
	Total	118.329	14			

Significant values are in bold.

Where SS is the sum of squares due to the source; df is the degrees of freedom in the source; MS is the mean sum of squares due to the source; F means the ‘F-statistic’.

**Figure 7:**
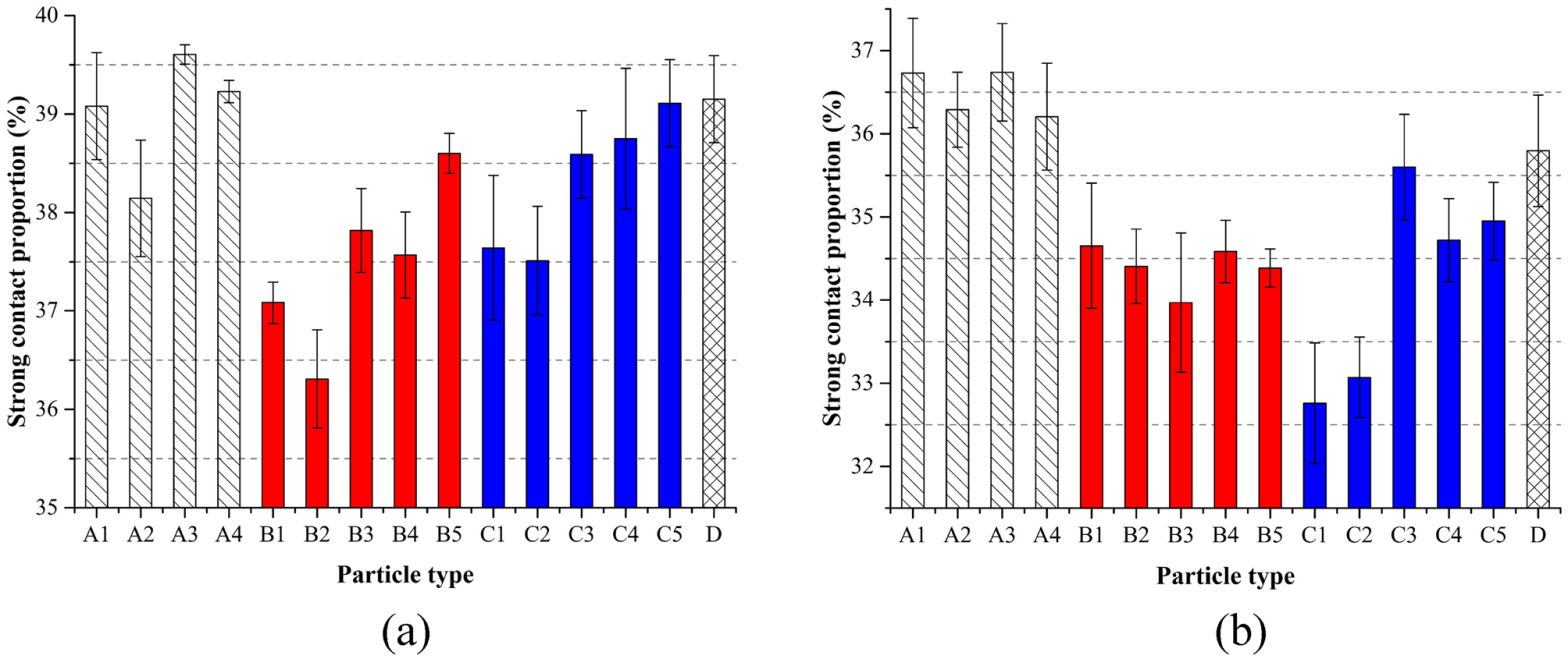
Strong contact proportions: (**a**) initial state; (**b**) peak state.

The interparticle contact network can be classified into two group: ‘strong’ contact and ‘weak’ contact^16,47^. When a normal contact force between two particles is lower than the average value, the contact belongs to a weak contact. On the contrary, the contact belongs to a strong contact. Strong contacts serve as a solid-like backbone, which could bear and transfer loads, whereas weak contacts provide stability against forces propagating through strong contacts^48^. It has been reported that the shear stress in granular materials is largely determined by the contributions of the strong contact network^47^. The shear stress within a granular assembly is largely attributed to the contribution of the strong contact network^47^. Figure 7 shows the strong contact proportions of different ballast assemblies at the initial state and the state when the peak stresses were obtained. At the initial state, the effect of shape parameters of A on the strong contact proportion cannot be clearly addressed. The proportion was around 39%. For assemblies B and C, with the increase of CON, strong contact proportions generally increased. However, the mean values of the strong contact proportions in assemblies B and C were lower than the mean value of the strong contact proportions in the assemblies A. When the shear stresses got the maximum values, the strong contact proportions decreased for all assemblies. The mean value of the assemblies A was significantly larger than those in assemblies B and C. However, for a single type of ballast, the variation of CON or RC could not lead to a clear trend on the change of the strong contact proportions.

**Figure 8:**
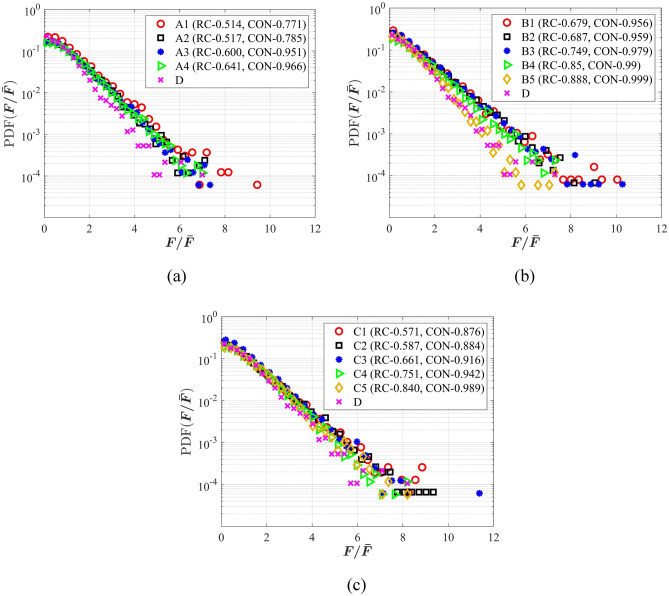
PDFs of contact force *F* normalized by the average $\overline{F}$ at the initial state: (**a**) assemblies of A and D; (**b**) assemblies of B and D; (**c)** assemblies of C and D.

To characterize the contact force distributions more clearly, the probability distribution functions (PDF) of the normal contact force (***F***) normalized by the mean normal contact force ($\overline{{\varvec{F}}}$) for all the assemblies were plotted in Fig. 8. When ${\varvec{F}}$/$\overline{{\varvec{F}}}$ ≤ 1.5, PDF(${\varvec{F}}$/$\overline{{\varvec{F}}}$) of the particle assemblies did not present significant differences. When 1.5 < ${\varvec{F}}$/$\overline{{\varvec{F}}}$ ≤ 6, PDF(${\varvec{F}}$/$\overline{{\varvec{F}}}$) of spherical particles (D) were significantly lower than those of particles A, B and C. It should be noted that when particles’ shape is closer to the spherical particle, the probability distributions are closer. In Fig. 8a, the probability distribution functions (PDF) of A4 and D were closer; while for types B and C, the probability distribution functions (PDF) of B5, C5 and D were closer. When ${\varvec{F}}$/$\overline{{\varvec{F}}}$ > 6, the contact forces of the complicated particles could achieve very large values. For particle A, the maximum value of ${\varvec{F}}$/$\overline{{\varvec{F}}}$ was 9.42; for particle B, the maximum value of ${\varvec{F}}$/$\overline{{\varvec{F}}}$ was 10.27; and for particle C, the maximum value of ${\varvec{F}}$/$\overline{{\varvec{F}}}$ was up to 11.37. This indicated that compared to spherical particles (D), the discreteness of the normal contact forces of particles A, B and C was larger, which was ascribed to complicated to the particle shapes.

**Figure 9:**
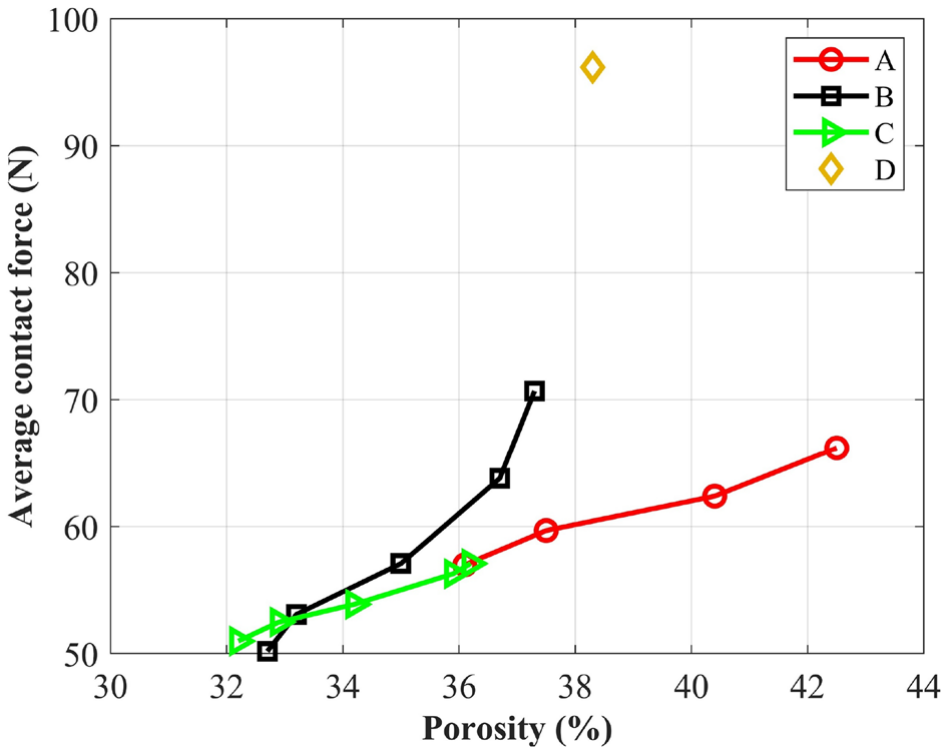
Relationship between porosity and the average contact force.

Figure 9 shows the relationship between porosity and the average contact force between particles. Clear trend can be observed that for particle A, B and C, with the increase of porosity, the average contact force increased significantly. Yu et al.^49^ reported that for a given type of particle, porosity is a function of interparticle force; a larger porosity generally induced a higher interparticle force. For particle D, the interparticle force was larger than those of particle A, B and C, which was caused by the difference in shape characteristics.

### Shear performance

**Figure 10:**
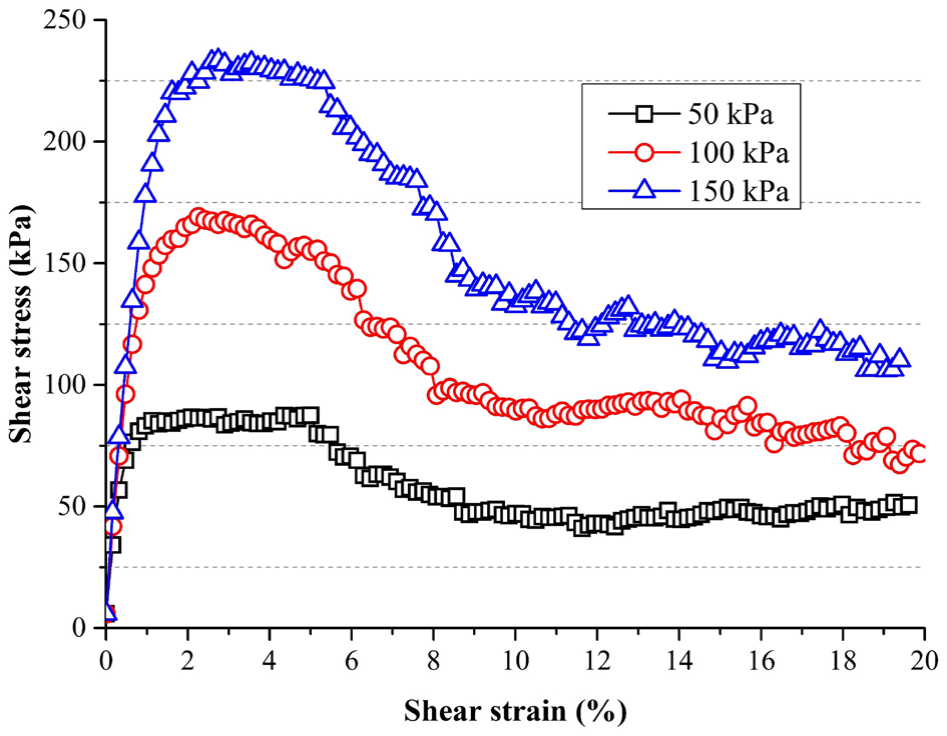
Shear stress–strain curves of samples B4.

**Figure 11:**
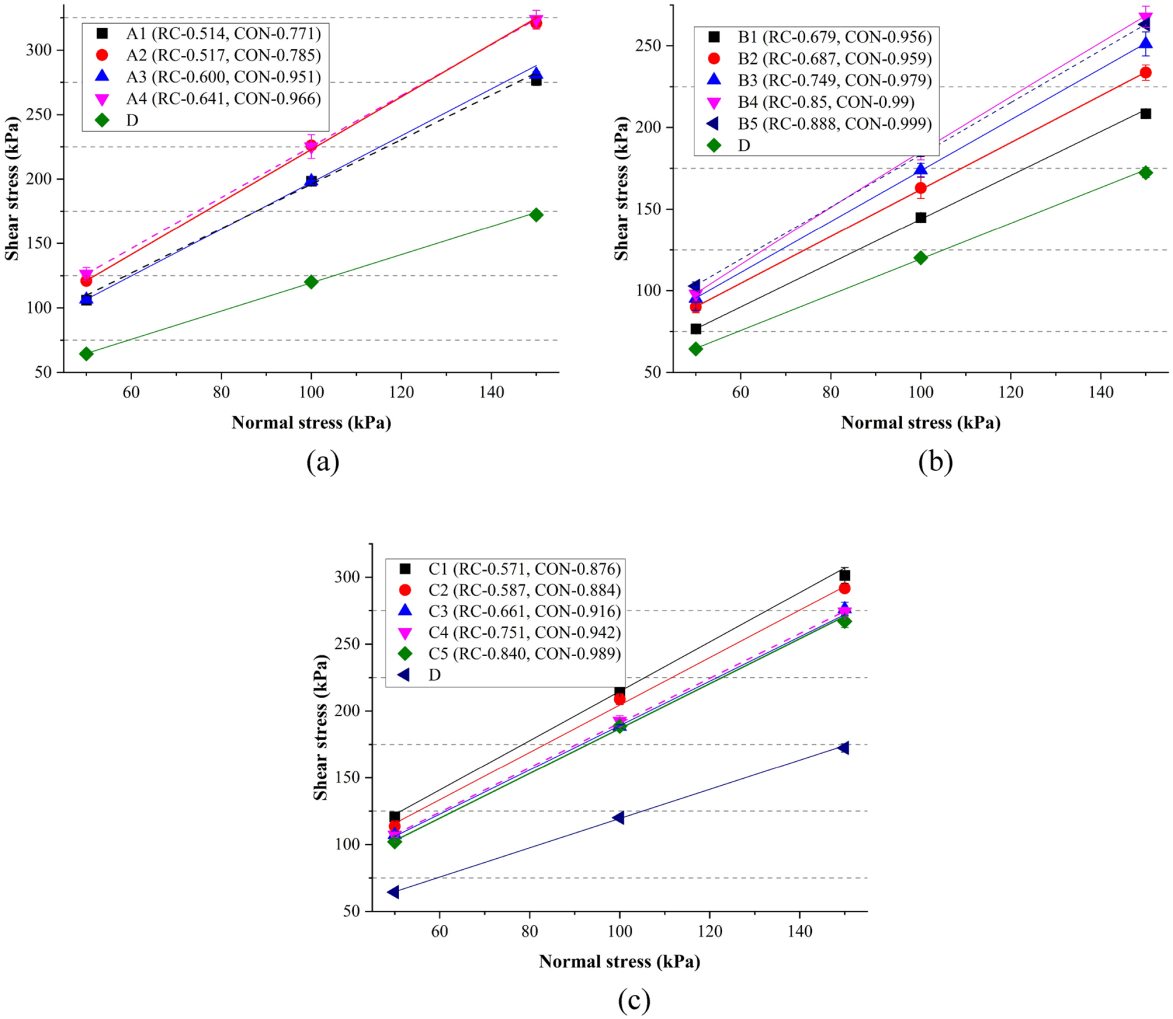
Normal stress versus shear stress: (**a**) assemblies of A and D; (**b**) assemblies of B and D; (**c**) assemblies of C and D.

Taking sample B4 as an example, the stress–strain behaviors were presented in Fig. 10. The shear strain is defined as the ratio between the displacement of the underlying box and the length of the box. After obtaining the peak shear stresses, Fig. 11 was plotted to show the relationship between normal stresses and shear stresses. Linear regression was performed according to the Mohr–Coulomb failure criterion. The cohesion strength (C) and the internal friction (tanφ) were shown in Table 4. It can be observed that the value of tanφ of spherical particles (D) was 1.10. For other complicated particles, values of tanφ were in the range of 1.34–2.04. Bian et al.^23^ conducted some DEM tests considering the angularity index and flat-elongated ratio of ballast particles. The values of tanφ are in the range of 1.47 and 2.53 for particles with different morphologies. Jing et al.^45^ found the values of tanφ changed from 1.28 to 2.14 correspondingly. The results of this study agree with the published results^23,45^.

**Table 4:** Parameters generated from Mohr–Coulomb law.

	A1	A2	A3	A4	B1	B2	B3	B4	B5	C1	C2	C3	C4	C5	D
C	23.48	19.20	15.96	27.55	9.60	18.19	16.80	12.90	23.39	30.19	27.14	13.15	23.85	18.98	10.02
tanφ	1.73	2.04	1.85	1.88	1.34	1.44	1.57	1.69	1.60	1.85	1.77	1.66	1.67	1.68	1.10

**Figure 12:**
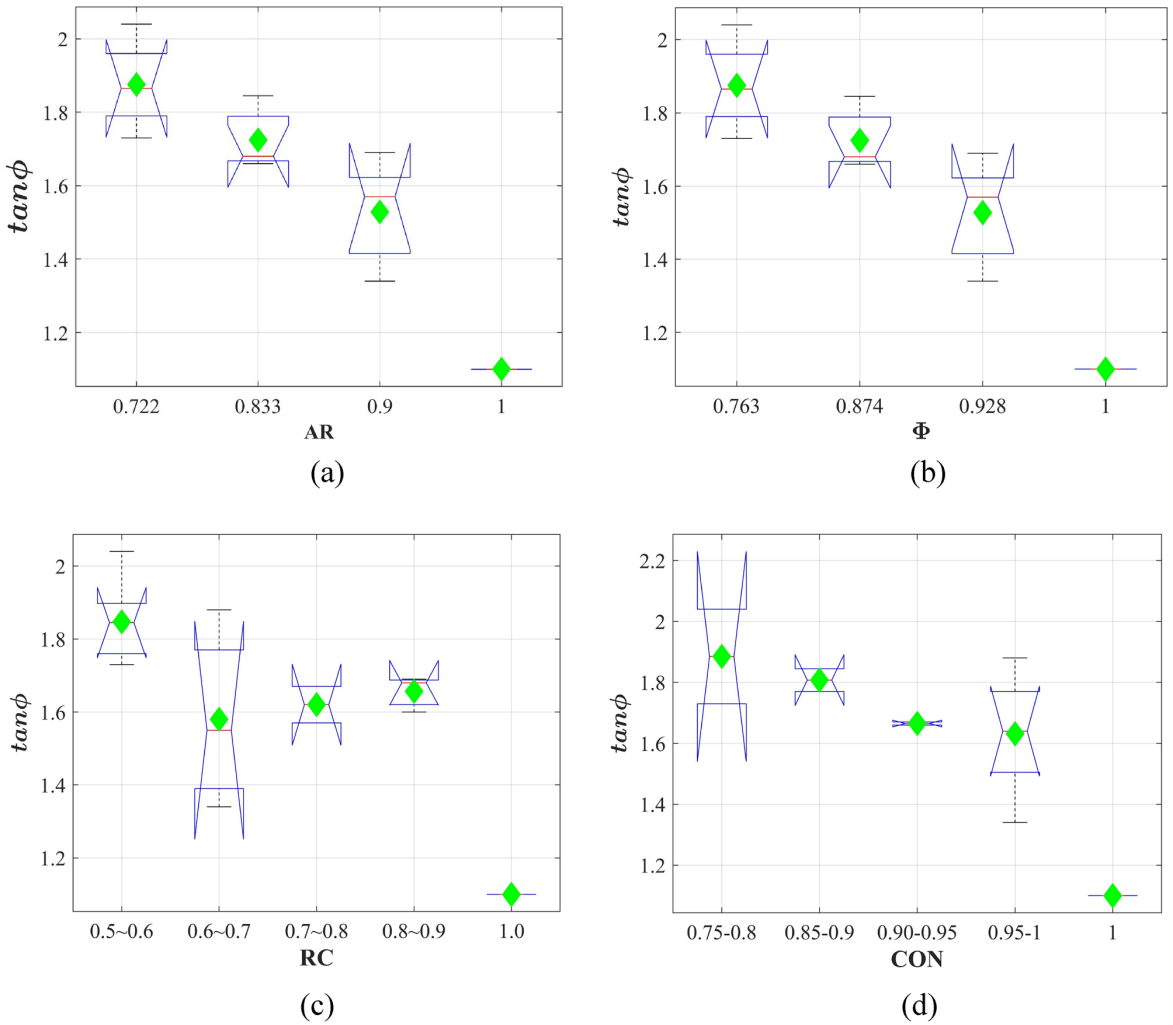
tanφ distributions for different parameters: (**a**) AR; (**b**) Φ; (**c**) RC; (**d**) CON.

In Table 4, the effects of different morphological parameters on the shear behaviors cannot be clearly observed. On the other hand, ballast particles own different morphological parameters. The simple comparison among the values in Table 4 cannot give accurate results. Therefore, some statistical techniques were employed to analyze the effect of particle shape on the shear behaviors. tanφ distributions for different parameters (AR, Φ, RC, and CON) were presented in Fig. 12. For each type of ballast, AR was constant, so as to Φ. Therefore, tanφ distributions are same for parameters AR and Φ. From Fig. 12a,b, it can be observed that with the increase of the aspect ratio (AR) and the sphericity (Φ), the internal friction effect in ballast assemblies become less significant. From Fig. 12c,d, simple conclusions can be made that with the increase of roundness (RC) and convexity (CON), magnitudes of tanφ decreased remarkably, indicating the interlock effect among particles become less significant. It should be noted that in Fig. 12c, when RC increased from the interval 0.6–0.7 to the interval 0.8–0.9, tanφ values slightly increased. However, when RC increased from 0.5 to 1.0, values of tanφ presented an overall downward trend. Moreover, one-way ANOVA tests were conducted to check the significance of each factor on the internal friction in direct shear tests. Table 5 shows the one-way ANOVA results of tanφ values. All parameters (AR, Φ, RC, CON) were significant factors at the 95% confidence interval.

**Table 5:** One way ANOVA tests of tanφ.

	Source	SS	df	MS	F	Prob > F
AR	Groups	0.661	3	0.202	14.74	**0**
	Error	0.151	11	0.014		
	Total	0.758	14			
Φ	Groups	0.661	3	0.202	14.74	**0**
	Error	0.151	11	0.014		
	Total	0.758	14			
RC	Groups	0.517	4	0.129	5.38	**0.014**
	Error	0.24	10	0.024		
	Total	0.758	14			
CON	Groups	0.467	4	0.117	3.97	**0.035**
	Error	0.293	10	0.029		
	Total	0.758	14			

Significant values are in bold.

**Figure 13:**
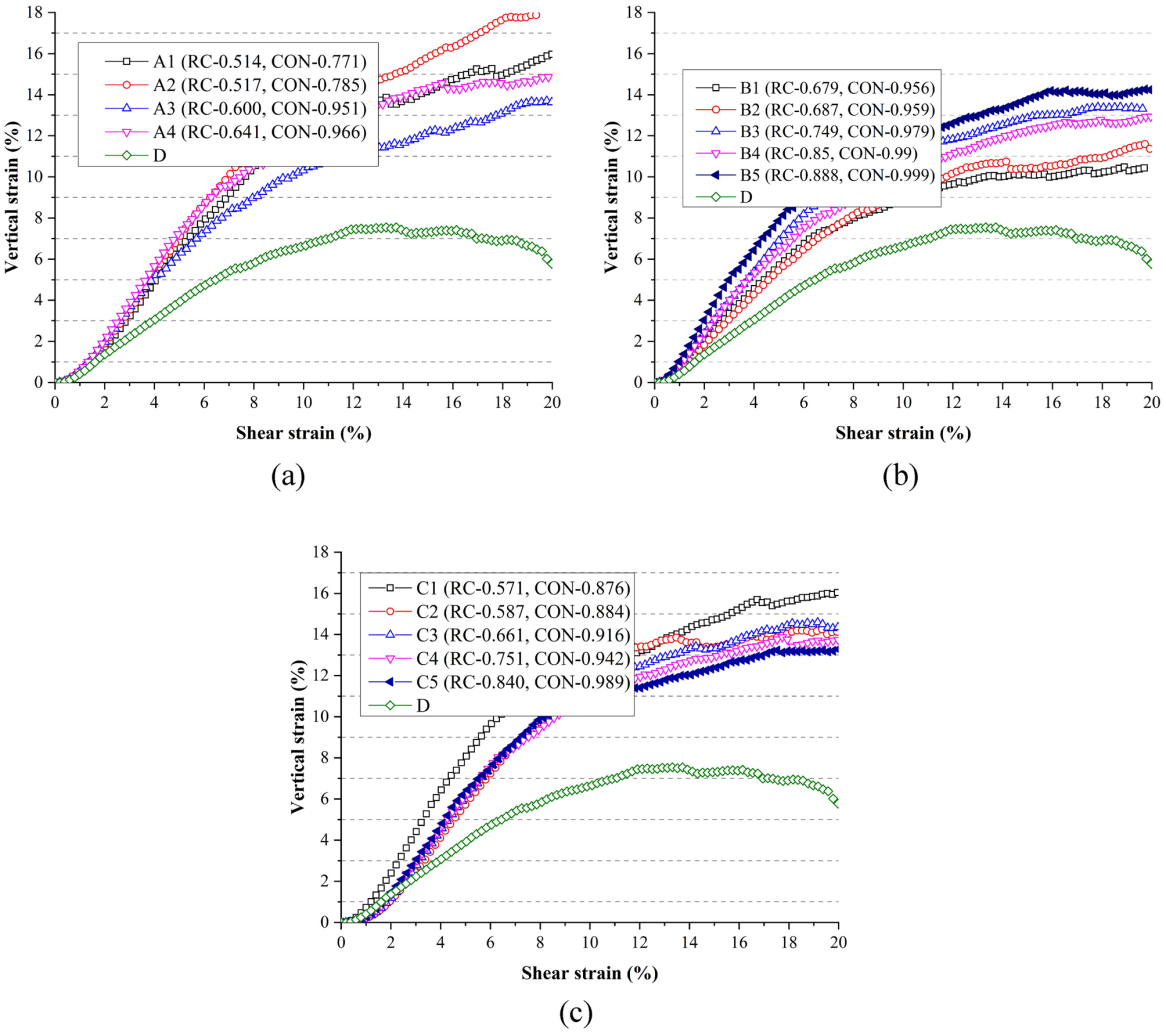
Shear strain vs. vertical strain: (**a**) assemblies of A and D; (**b**) assemblies of B and D; (**c**) assemblies of C and D.

The evolutions of vertical strains for all samples compressed under the normal stress of 150 kPa were monitored. Figure 13 presents the development of vertical strains along with the shear strains. A clear trend can be observed that when ballast particle evolved from a spherical particle to a complicated one, the vertical strain increased significantly. The vertical strain at 20% shear strain closely related to the initial compaction states of the assemblies. However, some general results can be derived that when the internal friction effect was most significant, the vertical strain was generally the largest. This is because the larger the internal friction, the more obvious the interlock effect in the shear band, and particles’ rotation may be more obvious. For particle A, A2 assembly gave the largest vertical strain, corresponding to the greatest tanφ value. The same statement existed for C1 assembly. For particle B, although the most significant dilatancy effect can be achieved for B5 assembly, which did not present the largest tanφ value. However, the vertical strains of assemblies B3, B4 and B5 did not present significant difference, tanφ values of assemblies B3, B4 and B5 were larger for particle B. For particle A, the vertical strain was in the range of 13.6–17.9%; for particle B, the range was 10.4% to 14.3%, and 13.3% to 16.1% for particle C. Therefore, with the increase of AR or Φ, vertical strains at the final state decreased, indicating the dilatancy effect become less obvious. For particle B and C, the vertical deformations reached to a stable state due to the constant vertical strains at the final state. However, for particle A, vertical strains still increased obviously at the final state.

Coordination number (CN) is the total number of contacts divided by the number of particles. CN is a very important parameter in DEM analysis to quantify the internal structure characteristics. For granular packing, the effective stress is transmitted through particle contacts. The calculation of CN should excludes ‘rattlers’ with zero or one contact^50^. The coordination number is defined as $CN = \left( {2N_{c} - N_{1} } \right)/\left( {N_{p} - N_{0} - N_{1} } \right)$ (where *N*_*c*_ is the total number of contacts, *N*_*1*_ is the number of particles which have just one contact, *N*_*0*_ is the number of particles which has no contact with other particles, and *N*_*p*_ is the total number of particles).

**Figure 14:**
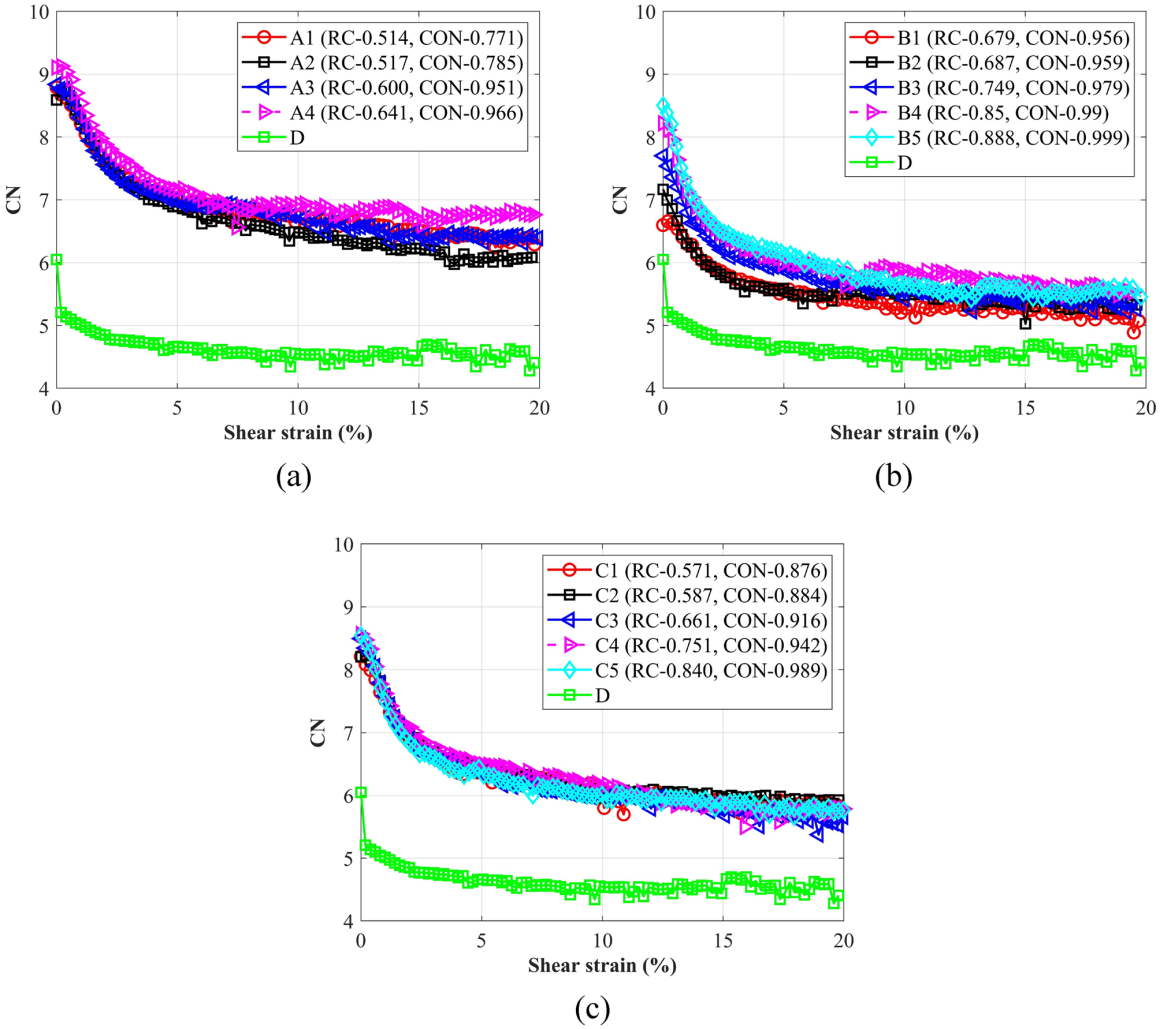
Coordination numbers: (**a**) assemblies of A and D; (**b**) assemblies of B and D; (**c**) assemblies of C and D.

**Figure 15:**
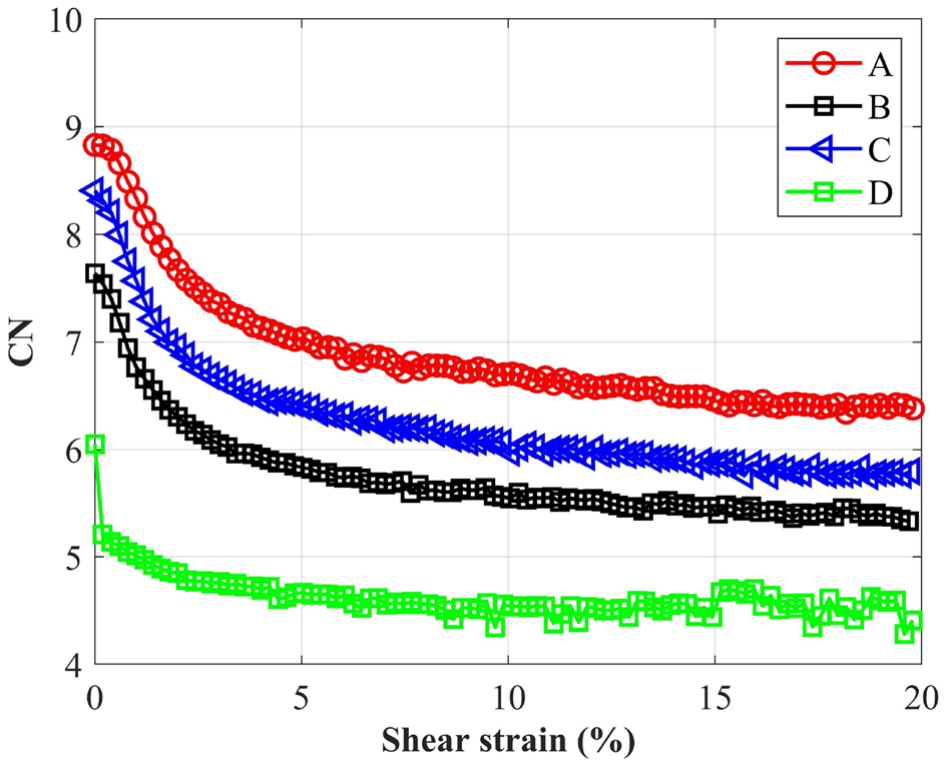
Average CNs of four types of particles.

It can be seen from Fig. 14 that at the shear beginning, CN decreased quickly; then CN slowly reached an almost constant value at the critical state. The initial rapid decrease of CN is attributed to the drastic perturbation of the packing state; the volume increase caused by the dilatancy also contributed to the CN decrease. At the same shear strain, CN of the spherical particle (D) were the smallest. For each type of ballast, there is no clear trend to demonstrate the effect of roundness (RC) or convexity (CON) on the evolution of CNs. It was reported^23^ that the evolution of CN not only depended on the particles’ shape, also on the initial porosities of the assemblies. A larger porosity generally induces a lower CN. According to Fig. 5, the porosity of A4 was the least, the same as B4 and B5 in type B assemblies. Thus, CN of A4 was larger than other packings in type A ballast, and CNs of B4 and B5 were also larger. To more clearly explore the impact of particle shape on CN, the average values of CN of particles A, B and C were derived and plotted in Fig. 15. It can be observed that with the increase of aspect ratio (AR) and sphericity (Φ), CN decreased significantly at the same shear strain, which was generally consistent with Bian et al.^23^. A larger AR or Φ means the particle is closer to a spherical particle, thus the contact states among particles were more similar with the states in spherical packings. On the other hand, as clump A is composed of 3 particles, clumps B and C only contain 2 particles, the irregular surface may induce more contact for particle assemblies A. For B and C, although the clump shape is similar, the difference in shape parameters led to the difference in CN.

## Conclusions and recommendation

In this study, direct shear tests were performed to explore the impacts of ballast shape by employing DEM. Four shape parameters, aspect ratio (AR), sphericity (Φ), roundness (RC) and convexity (CON), were obtained for four types of ballast. The initial packing states and the shear behaviors were quantitatively analyzed. One-way ANOVA tests were performed to investigate the significance of each parameters on the initial packing states and the shear behaviors. Some main conclusions can be drawn below:

1. AR, Φ, RC and CON were all significant in affecting the initial porosities. The initial porosity decreased first and increased subsequently with the increase of each parameter. When the minimum porosities were obtained, the corresponding AR, Φ, RC and CON were 0.9, 0.928, 0.80–0.90 and 0.90–0.95, respectively.
2. When ${\varvec{F}}$/$\overline{{\varvec{F}}}$ ≤ 1.5, PDF(${\varvec{F}}$/$\overline{{\varvec{F}}}$) of all particle assemblies did not present significant differences. When 1.5 < ${\varvec{F}}$/$\overline{{\varvec{F}}}$ ≤ 6, PDF(${\varvec{F}}$/$\overline{{\varvec{F}}}$) of spherical particles (D) were significantly lower than those of particles A, B and C. When ${\varvec{F}}$/$\overline{{\varvec{F}}}$ > 6, the contact forces of the complicated particles could achieve very large values, indicating the discreteness of the normal contact forces of particles A, B and C was larger.
3. The relationship between the normal stress the peak shear stress can be characterized by the Mohr–Coulomb failure criterion. AR, Φ, RC and CON were all significant factors in affecting the internal friction. When the increase of each parameter, the internal friction decreased obviously and the internal friction inside the spherical ballast assemblies was the lowest.
4. With the increase of AR or Φ, the coordination number (CN) decreased at the same shear strain. This was ascribed to the fact that a larger AR or Φ meant the particle was closer to a spherical particle, and thus the CN became smaller.
5. The ballast in this study were generated based on the numerical method. For real ballast particles, the effects of the shape parameters on the direct shear performance would be explored in the future study. On the other hand, DEM tests based 3D aggregate shape would be conducted in future.

## Data Availability

The datasets used during the current study are available from the corresponding author on reasonable request.

## References

[1] Lim WL (2004). Mechanics of railway ballast behaviour.

[2] Shi C, Zhao C, Zhang X, Guo Y (2021). Coupled discrete-continuum approach for railway ballast track and subgrade macro-meso analysis. Int. J. Pavement Eng..

[3] Song W (2019). Improving damping properties of railway ballast by addition of tire-derived aggregate. Transp. Res. Rec..

[4] Wu H (2021). Impact performance of ballast by incorporating waste tire-derived aggregates. Constr. Build. Mater..

[5] Shi C (2023). Railway ballast performance: recent advances in the understanding of geometry, distribution and degradation. Transp. Geotech..

[6] Khatibi F, Esmaeili M, DEM Mohammadzadeh S (2017). analysis of railway track lateral resistance. Soils Found..

[7] Hou W, Feng B, Li W, Tutumluer E (2018). Evaluation of ballast behavior under different tie support conditions using discrete element modeling. Transp. Res. Rec..

[8] AREMA. (American Railway Engineering and Maintenance-of-Way Association, Lanham, MD, 2010).

[9] MOR. (China Railway Press, Beijing, 2008).

[10] Huang H, Tutumluer E (2011). Discrete element modeling for fouled railroad ballast. Constr. Build. Mater..

[11] Ngo NT, Indraratna B, Rujikiatkamjorn C (2017). Micromechanics-based investigation of fouled ballast using large-scale triaxial tests and discrete element modeling. J. Geotech. Geoenviron. Eng..

[12] Guo Y, Markine V, Song J, Jing G (2018). Ballast degradation: Effect of particle size and shape using Los Angeles Abrasion test and image analysis. Constr. Build. Mater..

[13] Xu Y, Gao L, Zhang Y-R, Yin H, Cai X-P (2016). Discrete element method analysis of lateral resistance of fouled ballast bed. J. Central South Univ..

[14] Hu J, Ma T, Ma K (2021). DEM-CFD simulation on clogging and degradation of air voids in double-layer porous asphalt pavement under rainfall. J. Hydrol..

[15] Zhao S, Zhou X (2017). Effects of particle asphericity on the macro-and micro-mechanical behaviors of granular assemblies. Granul. Matter.

[16] Song W, Xu F, Wu H, Xu Z (2021). Investigating the skeleton behaviors of open-graded friction course using discrete element method. Powder Technol..

[17] Song W, Huang B, Shu X, Stránský J, Wu H (2019). Interaction between railroad ballast and sleeper: a DEM-FEM approach. Int. J. Geomech..

[18] Cundall PA, Strack OD (1979). A discrete numerical model for granular assemblies. Geotechnique.

[19] Gong J, Liu J (2017). Effect of aspect ratio on triaxial compression of multi-sphere ellipsoid assemblies simulated using a discrete element method. Particuology.

[20] Deng XL, Davé RN (2013). Dynamic simulation of particle packing influenced by size, aspect ratio and surface energy. Granul. Matter.

[21] Yang J, Luo XD (2015). Exploring the relationship between critical state and particle shape for granular materials. J. Mech. Phys. Solids.

[22] Jiang MD, Yang ZX, Barreto D, Xie YH (2018). The influence of particle-size distribution on critical state behavior of spherical and non-spherical particle assemblies. Granul. Matter.

[23] Bian X, Li W, Qian Y, Tutumluer E (2019). Micromechanical particle interactions in railway ballast through DEM simulations of direct shear tests. Int. J. Geomech..

[24] Xiao J (2020). Morphological reconstruction method of irregular shaped ballast particles and application in numerical simulation of ballasted track. Transp. Geotech..

[25] Nie Z, Zhu Y, Wang X, Gong J (2019). Investigating the efects of Fourier-based particle shape on the shear behaviors of rockfll material via DEM. Granul. Matter.

[26] Danesh A, Mirghasemi AA, Palassi M (2020). Evaluation of particle shape on direct shear mechanical behavior of ballast assembly using discrete element method (DEM). Transp. Geotech..

[27] Paixão A, Resende R, Fortunato E (2018). Photogrammetry for digital reconstruction of railway ballast particles–a cost-efficient method. Constr. Build. Mater..

[28] Guo Y (2020). Discrete element modelling of railway ballast performance considering particle shape and rolling resistance. Railw. Eng. Sci..

[29] Suhr B, Skipper WA, Lewis R, Six K (2020). Shape analysis of railway ballast stones: curvature-based calculation of particle angularity. Sci. Rep..

[30] Zhang Z-T, Gao W-H, Wang X, Zhang J-Q, Tang X-Y (2020). Degradation-induced evolution of particle roundness and its effect on the shear behaviour of railway ballast. Transp. Geotech..

[31] Lu M, McDowell G (2007). The importance of modelling ballast particle shape in the discrete element method. Granul. Matter.

[32] Liu L, Ji S (2020). A new contact detection method for arbitrary dilatedpolyhedra with potential function in discreteelement method. Int. J. Numer. Meth. Eng..

[33] Zhao S, Zhao J (2019). A poly-superellipsoid-based approach on particle morphology for DEM modeling of granular media. Int. J. Numer. Anal. Meth. Geomech..

[34] Wang S, Marmysh D, Ji S (2020). Construction of irregular particles with superquadric equation in DEM. Theor. Appl. Mech. Lett..

[35] Chandler R, Hamilton P (1999). On the measurement of the undrained strength of discontinuities in the direct shear box. Géotechnique.

[36] Sadeghi J, Kian ART, Ghiasinejad H, Moqaddam MF, Motevalli S (2020). Effectiveness of geogrid reinforcement in improvement of mechanical behavior of sand-contaminated ballast. Geotext. Geomembr..

[37] TolouKian AR, Sadeghi J, Zakeri J-A (2018). Large-scale direct shear tests on sand-contaminated ballast. Proc. Inst. Civ. Eng. Geotech. Eng..

[38] Mishra, D. & Mahmud, S. N. in *ASME/IEEE Joint Rail Conference.* V001T001A014 (American Society of Mechanical Engineers).

[39] Zhao B, Wang J (2016). 3D quantitative shape analysis on form, roundness, and compactness with μCT. Powder Technol..

[40] Wei D, Wang J, Zhao B (2018). A simple method for particle shape generation with spherical harmonics. Powder Technol..

[41] Cruz-Matías, I. & Ayala, D. Orientation, sphericity and roundness evaluation of particles using alternative 3D representations (2014).

[42] Indraratna B, Wijewardena L, Balasubramaniam A (1993). Large-scale triaxial testing of greywacke rockfill. Geotechnique.

[43] Suhr B, Marschnig S, Six K (2018). Comparison of two different types of railway ballast in compression and direct shear tests: experimental results and DEM model validation. Granul. Matter.

[44] Gong H (2019). Direct shear properties of railway ballast mixed with tire derived aggregates: experimental and numerical investigations. Constr. Build. Mater..

[45] Jing GQ, Ji YM, Qiang WL, Zhang R (2020). Experimental and Numerical study on ballast flakiness and elongation index by direct shear test. Int. J. Geomech..

[46] Kozicki J, Donze FV (2009). YADE-OPEN DEM: an open-source software using a discrete element method to simulate granular material. Eng. Comput..

[47] Radjai F, Wolf DE, Jean M, Moreau J-J (1998). Bimodal character of stress transmission in granular packings. Phys. Rev. Lett..

[48] Gong J, Liu J, Cui L (2019). Shear behaviors of granular mixtures of gravel-shaped coarse and spherical fine particles investigated via discrete element method. Powder Technol..

[49] Yu A, Feng C, Zou R, Yang R (2003). On the relationship between porosity and interparticle forces. Powder Technol..

[50] Thornton C (2000). Numerical simulations of deviatoric shear deformation of granular media. Géotechnique.

